# Tics induced by promethazine in an adolescent: a case report

**DOI:** 10.1192/bjo.2021.332

**Published:** 2021-06-18

**Authors:** Fiyinfoluwa Akinsiku

**Affiliations:** Herefordshire and Worcestershire Health and Care Trust

## Abstract

**Objective:**

The objective of this report is to highlight the finding of a movement disorder caused by promethazine in a 16-year-old female and to alert other clinicians to a high index of suspicion of possible movement disorders in young people on promethazine.

**Case report:**

I discuss a 16-year-old female (who presented to medics at 15) with low mood, lack of motivation, self-consciousness – at 15, she was over 6 feet tall and weighed 81.2 kg. She also self-harmed by cutting her thigh with razor along with poor sleep, anxiety, and panic attacks. She took an overdose of paracetamol and ibuprofen and a strip of vitamin D and irritable bowel tablets she found at home.

A clinical impression of moderate depression with anxiety and panic attacks and possible emerging emotionally unstable personality traits was made and she had begun psychological sessions with the therapist before referral to the medics. Fluoxetine 20 mg OD increased to 40 mg and Circadin 2 mg ON was commenced. Fluoxetine was later tapered off and Circadin stopped. Sertraline 100 mg OD increased to 200 mg was commenced and Promethazine 25 mg ON to improve sleep.

Within a month of commencement of promethazine, a sudden onset of extension of neck, blowing through lips and a high-pitched sound occurred whilst experiencing a panic attack and hyperventilating. She also stuttered and had difficulty in speaking, and her vision would go blurry. She initially refused to come off promethazine as it had helped her sleep. An impression of a tic disorder characterised by motor and vocal tics was made. There had been no recent infections or previous history or family history of tics. However, at this point, sertraline had helped with her motivation and she was able to come off promethazine and her sleep was improved by practising sleep hygiene with an accompanied cessation of tics.

**Discussion:**

Young person is currently on 150 mg of Sertraline.

The rationale behind reporting this case is that previous studies have pointed at SSRIs, as causes of tics disorders, but promethazine is one that does a good job in improving sleep and has a side effects of movement disorder.

**Conclusion:**

Promethazine is one medication that can cause movement disorder and a high index of suspicion coupled with a prompt cessation of medication will reduce patient's distress and improve the therapeutic relationship between health professional and young person.

Written informed consent from patient and guardian was got.

Author declares that there is no conflicting interest, financial or otherwise.

